# Effects of different tea tree varieties on the color, aroma, and taste of Chinese Enshi green tea

**DOI:** 10.1016/j.fochx.2022.100289

**Published:** 2022-03-22

**Authors:** Yuchuan Li, Wei Ran, Chang He, Jingtao Zhou, Yuqiong Chen, Zhi Yu, Dejiang Ni

**Affiliations:** a Key Laboratory of Horticulture Plant Biology, Ministry of Education, College of Horticulture & Forestry Sciences, Huazhong Agricultural University, Wuhan, Hubei 430070, People’s Republic of China; b Key Laboratory of Urban Agriculture in Central China, Ministry of Agriculture, Wuhan, Hubei 430070, People’s Republic of China

**Keywords:** Enshi green tea, Color, Aroma, Taste, Echa 10

## Abstract

Green tea processed by Echa 10 was shown to have a fresh and mellow taste as well as clean aroma with a clear honeysuckle fragrance. The colors of different Enshi green teas are closely related with the content of chlorophyll and chlorophyllide. The five green teas also vary in their aroma style. Echa 10 imparts a special honeysuckle fragrance, which was further analyzed by molecular sensory analysis and the formation of this honeysuckle fragrance was attributed to the key components of dodecane, octadecane, phenethyl alcohol, and jasmonone. In aroma evaluation, Echa 10 green tea showed the best performance, which is mainly related with the content of geraniol, linalool, phenethyl alcohol, and benzyl alcohol. Additionally, Echa 10 scored the highest in taste evaluation, which is mainly determined by the contents and ratios of tea polyphenols, amino acids, caffeine, and soluble sugars.

## Highlights

• Chinese Enshi green tea quality varies with tea tree varieties.
• Chlorophyll and chlorophyllide determine the green tea color.
• Echa 10 endows Enshi green tea with fresh and mellow taste.
• Echa 10 endows Enshi green tea with clear flavor and honeysuckle fragrance.
• Phenethyl alcohol, jasmine, dodecane and octadecane contribute to honeysuckle scent.

## Introduction

Green tea, a global beverage, is widely produced in China, Japan, Vietnam, and other countries, with the largest production volume in China (Guo, Ho, Schwab & Wan, 2021). Many previous studies have shown that the quality of green tea is greatly affected by environment, variety, cultivation, and processing technology, with variety being considered as the main factor affecting its color, aroma, and taste (Chen et al., 2021, Wang et al., 2021, Xu et al., 2021).

The quality of green tea is mainly determined by color, fragrance, taste, and shape, namely green color, fresh fragrance with floral (or chestnut) aroma, fresh and mellow taste, and compact shape (Chen et al., 2021). The quality of tea is closely related to the variety of tea trees (Wang, Cao, Yuan & Guo, 2021). Studies have shown that the strong taste and red color of black tea are usually related to the formation of a large amount of theaflavins during the processing of large tea leaves from Yunnan Daye variety (Li et al., 2021).The aroma quality of oolong tea is related to the release of aroma glycosides in the leaves of Tieguanyin variety during the shaking process (Hu et al., 2018). The quality of green tea is also affected by the variety of tea trees. Longjing 43 was reported to be the most suitable variety for processing West Lake Longjing tea, mainly because of its high content of chlorophyll *b*, contributing to the formation of brown beige in dry tea (Wang & Ruan, 2010). The Taiping Houkui tea made from the shidacha variety has a strong orchid fragrance, which is related to the formation of phenotype methyl jasmonate in the fresh leaves during processing (Feng, Li, Li, Wan & Yang, 2019). Anji bai tea processed from Baiye 1 has a fresh and mellow taste, which is related to the high content of l-theanine and low tea polyphenols (Zeng, Lin, Liu & Liu, 2019). In China, the provinces to the south of the Yangtze River Basin all produce green tea. Due to environmental differences caused by geographical location, tea varieties vary in their adaptation, leading to changes in the connotative components during the growth process and thus different qualities for the same green tea processed from the same raw materials (Wang et al., 2021, Xu et al., 2021). However, to our knowledge, no in-depth study has been performed on the quality differences between different varieties of green tea made by the same processing method.

The main production area of Enshi green tea is in Enshi Prefecture, Hubei Province, China, with a planting area of about 0.133 million hectare, and the tea industry has become the main source of income for tea farmers. Due to its good quality, the tea has become the best-selling tea brand in Hubei, China (Dong et al., 2018). Our preliminary investigation found that Enshi area has a fairly large number of tea tree varieties, resulting in uneven quality of finished tea and affecting the economic benefits of local tea farmers. In this study, we selected five tea tree varieties mainly planted in this area, and used the same processing method to make Enshi green tea. The purpose of this study was to investigate the effects of different varieties on the quality of color, aroma, and taste of the green tea by chromatography, mass spectrometry and other related methods, with a focus on the use of gas chromatography-mass spectrometry-olfactometry (GC–MS-O) to reveal the fragrance and floral aroma of Enshi green tea processed from the special variety Echa 10. This study may provide a theoretical basis for the quality improvement of Enshi green tea and the breeding of high-quality tea varieties.

## Materials and methods

### Main chemicals

The main chemicals used in this study included ninhydrin, anthrone, concentrated sulfuric acid, and acetone (AR, China national pharmaceutical group, Shanghai chemical reagent Co., Ltd.); Folinphenol (AR, Sigma, USA); methanol and ether (AR, Fisher Scientific, Ireland); chlorophyll *a*, and chlorophyll *b* (standard products, Sigma-Aldrich, USA); lutein and β-carotene (standard products, Shanghai Yuanye Biotechnology Co., Ltd.).

### Processing of tea samples

In April 2019, fresh tea leaves (one bud with one leaves) were picked in the base of Wutai Changchen Tea Industry Co., Ltd. in Xuan'en, Hubei. Five varieties (Echa 1, Echa 10, Zhenong 117, Mingshan 131 and Fuyun 6) were selected in this study, and all the tea trees were 7–8 years old. The green teas were made using the same processing procedure: fresh leaves → withering (spreading fresh leaves at 2–3 cm thickness, 6 h) → fixation (type 80-A electromagnetic drum fixation machine, 240 °C) → cooling and moisturizing for 2 h → rolling (type 6CR-65 tea rolling machine, 40 min) → initial drying (DF-7 type hot air shaping platform, 65 °C, 20 min) → cooling and moisturizing (20 min) → shaping (6CLZ-80 famous tea stripping machine, temperature 190 °C, 6 min) → cooling and moisturizing (60 min) → final drying (DF-7 type hot air shaping platform, 65 °C). All the above experiments were repeated 3 times.

### Characterization of green tea samples

Sensory evaluation was performed independently by 5 professional tea tasters on a 100-point scale: appearance of dry tea (25%), brew color (10%), aroma (25%), taste (30%), and infused leaf (10%) (Yu et al., 2019).

Aqueous extract was characterized according to the National Standard of China (GB/T 8305–2013) (Qu, Zhu, Ai, Ai, Qiu, & Ni, 2019). Tea polyphenol content was measured by using the colorimetric method; amino acid content was quantified using the GB/T8314-2013 method; soluble sugar content was measured by the anthrone-sulfuric acid colorimetric method (Li et al., 2021). Caffeine was analyzed by HPLC (Qu, Zhu, Ai, Ai, Qiu, & Ni, 2019). Chlorophyll was extracted by acetone trituration and analyzed by HPLC (Yu et al., 2019). The color quality of dry tea and tea infusion was analyzed using the colorimeter method (Yu et al., 2020).

### Extraction and analysis of volatile compounds in green tea samples

The aroma in different green tea samples was determined by headspace solid phase extraction-GC–MS (Li et al., 2022). For extraction of aroma components, the PDMS/DVB extraction fibers were aged at 250 °C for 1 h at the GC injection port. Next, each crushed tea sample (1.0000 g) was weighed into a 20 mL headspace bottle, followed by adding 5 mL of boiling distilled water to brew, then adding 200 μL of ethyl caprate internal standard solution (0.1 μL/100 mL), and finally adsorption for 60 min in a 60 °C water bath. GC–MS analysis was performed under the following conditions: inlet temperature 230 °C; carrier gas, high-purity helium (≥99.99%); column flow rate, 1.0 mL/min; heating program: initial temperature 40 °C, hold for 2 min, up to 85 °C at 5 °C/min, hold for 2 min, up to 110 °C at 2 °C/min, up to 130 °C at 7 °C/min, up to 230 °C at 5 °C/min, and hold for 8 min; oven temperature, 40.0 °C; injection mode, splitless. MS conditions: ion source, EI; electron energy, 70 eV; ion source temperature, 230℃; mass scanning range, 35 ∼ 400 *m*/*z*. Substance was characterized by RI value, NIST2014 database, and retention time.

### Isolation of volatiles by solvent-assisted flavour evaporation (SAFE)

Essential oils were extracted from tea samples as previously reported (Xu et al., 2021). Briefly, 500 mL of boiled distilled water was added to 10 g of tea leaves, followed by soaking for 10 min, filtering out the tea residues, cooling the tea soup to room temperature, and adding the tea soup into the SAFE distillation equipment at the indicated amount. Next, the solid solution was obtained in the collection bottle, followed by thawing it with running water and adding 3 g of sodium chloride to facilitate the separation of the organic phase. Subsequently, 20 mL of dichloromethane (chromatographically pure) was added into the collection system, followed by ultrasonic extraction in an ice-water bath for 20 min, and separating the organic phase and the water phase. This extraction process was repeated four times, and the separated organic phase was dried over anhydrous sodium sulfate. After standing still for several hours until the removal of water, the solution was filtered and seal stored at −20℃ for further analysis. Before smell analysis, the obtained solution was distilled to 10 mL with Vigreux column at 40 °C and brought to 0.5 mL by blowing nitrogen through it in a mild nitrogen ice bath. The resulting solution was used for subsequent smell experiments.

### Aroma extract dilution analysis and GC–MS-O analysis of green tea concentrate samples

The green tea concentrate was diluted serially with dichloromethane at the volume ratio of 1:4, 1:16, 1:32, 1:64, and 1:256. When the evaluator could no longer detect the odour at the end of the sniffing mouth, dilution was stopped and the flavour dilution factor (FD) was defined as the highest dilution ratio of the corresponding substance detectable by the evaluator. The flavour evaluation was performed by 4 panelists, and each panelist was required to record the perceived odour, describe the odour characteristics, and record the retention time. Compounds were identified by matching the retention index, odour description, and mass spectrum of the unknown compound to the retention index of its reference counterpart.

GC–MS analysis of green tea samples was performed under following conditions: Thermo Scientific ISQ 7000 mass spectrometer system, equipped with TRACE 1300 chromatography system (chromatographic column: TG-WAXMS A 60 m*0.25 mm*0.25um capillary column). Gas, high purity helium; carrier gas flow rate: 1 mL/min; gradient heating program: the initial temperature 40 °C, hold for 2 min, up to 230 °C at 5 °C/min, and hold for 15 min; injection amount, 1 µL; injection mode, splitless; ion source, EI; electron energy, 70 eV; mass interface temperature, 260 °C; ion source temperature, 280 °C; mass scanning range, 45–400 *m*/*z*.

### Statistical analysis

The results were provided as means ± standard deviation (SD) (n = 3), and statistically significant difference was considered at *P* less than 0.05. Data analysis was done with excel and SPSS software. Graphs were drawn by OriginPro 2018, Smica 14, and Adobe Illustrator CC 2019.

## Results and discussion

### Sensory evaluation

**Fig. 1:**
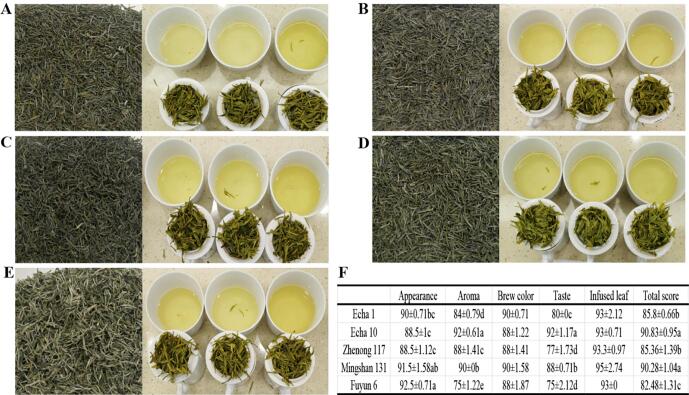
Sensory evaluation of five green teas. A: Echa 1, B: Echa 10, C: Zhenong 117, D: Mingshan 131, E: Fuyun 6, F: sensory score.

Fig. 1 shows the sensory evaluation results of five green teas. The five green teas were seen to vary mainly in the appearance color, aroma, and taste, but with no significant difference in brew color and infused leaves. In appearance color, Fuyun 6 showed the highest score (92.5), followed by Mingshan 131, Echa 1, Echa 10 and Zhenong 117, with the same greenness/score (88.5) for the latter two. In aroma evaluation, Echa 10 achieved the highest score (92) with a honeysuckle fragrance, followed by Mingshan 131 with a tender fragrance and a score of (90), Zhenong 117 with a fresh fragrance and a score of 88, Echa 1 with a score of 84 and a green grassy smell, and Fuyun 6 with a poor aroma score (75) due to a distinct aroma of roasted sweet potatoes. Generally, high-quality green tea is characterized by a fresh, floral and tender fragrance, while roasted sweet potato fragrance and green grassy smell do not meet the high-level green tea requirements, which may be related to the characteristics of varieties (Ho et al., 2015, Guo et al., 2021). For taste quality, Echa 10 exhibited the highest score of fresh and mellow (92), followed by Mingshan131 (88). Echa 1 (80) and Zhenong 117 (77) showed obvious astringency, and Fuyun 6 (75) exhibited obvious roasted sweet potato taste. Generally, the astringency of tea can be alleviated by the low-temperature aromatizing technique in the later drying stage, but the roasted sweet potato flavour cannot be easily changed, which is often determined by the characteristics of the variety (Qu et al., 2019, Fu et al., 2020). The aroma and taste evaluation results indicated that Fuyun 6 is not suitable for processing high-level green tea in this area. Additionally, brew color and infused leaf evaluation showed that all the five tea varieties can meet the color requirements for Enshi green tea production, but with a better green color for Mingshan 131 than others. For total score, Echa 10 Enshi green tea scored the highest (90.83), followed by Mingshan 131 (90.28), Zhenong 117 (85.33), Echa 1 (84.80), and Fuyun 6 (82.48). Based on sensory quality, Echa 10 and Mingshan 131 are two varieties suitable for Enshi green tea production, especially Echa 10 with an obvious honeysuckle fragrance.

### Effects of different tea tree varieties on the color of Enshi green tea

**Fig. 2:**
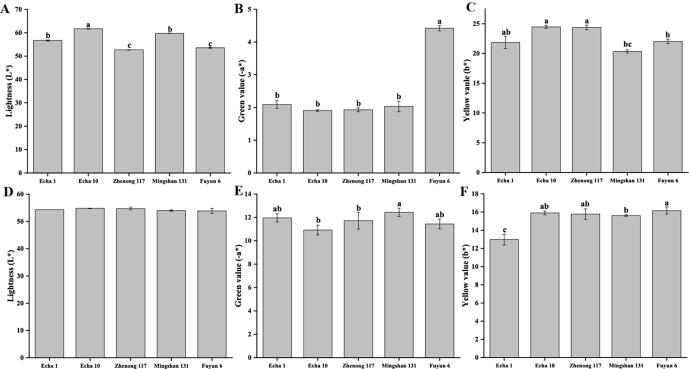
Chromatic aberration analysis of dry tea and tea soup of different green teas. A-C: color values of dry tea, D-F: color values of tea soup.

Fig. 2 shows the different color values of dry tea and tea soup of the 5 varieties, which varied significantly in the color values of dry tea, but with little difference in the color values of tea soup. The color quality was evaluated in terms of lightness(L*), greenness(-a*), and yellowness (b*). The larger the L* value, the higher the lightness, and the absolute values of a* and b* are positively related to their hue (Yu et al., 2020). Echa 10 was significantly higher than the other varieties in the L* value of dry tea, indicating its best lightness. Meanwhile, Fuyun 6 showed the highest absolute a* value of dry tea (-4.42), which was consistent with its sensory evaluation result (emerald green). Additionally, Echa 10 and Zhenong 117 exhibited higher b* values of dry tea, indicating the increase of yellowness and the decrease of greenness, which was also consistent with the sensory evaluation results. The difference of the three color values in tea soup is mainly reflected in b*. Specifically, Echa 1 was significantly smaller than the other varieties in the b* value of tea soup, but the five varieties showed little difference in L* and a* values, suggesting no significant difference among the five varieties in the tea soup color, which also agreed with the brew color evaluation results in Fig. 1**F**.

The color quality of tea is the result of the combined action of chlorophyll and its transformation products, with chlorophyll as the main substance for the color formation of green tea (Yang, Li, Teng, Han, & Zhuang, 2021). The difference of the five varieties in the color of dry tea was further explored by using HPLC to quantify the main components of chlorophyll and its degradation products. Previous studies have shown that the main substances for the color formation of green tea are chlorophyll *a* and chlorophyll *b*, which are blue-green and yellow-green, respectively (Yu et al., 2019). Table 1 shows the contents of chlorophyll in the five varieties of green tea. Fuyun 6 was seen to have a significantly higher content of chlorophyll *a* than the other varieties, and the content of chlorophyll *b* was in the order of Echa 1 > Echa 10 > Zhenong 117 > Fuyun 6 > Mingshan 131. Comparison of the other substances in the five varieties unveiled that Fuyun 6 was significantly higher than the other varieties in the relative content of chlorophyllide a and pheophorbide a. Meanwhile, Echa 10 and Echa 1 exhibited higher pheophytin content. Studies have shown that chlorophyll (Chl) and chlorophyllide (Cd) are mostly green, while pheophorbide (Po) and pheophytin (Py) are yellow–brown or dark-brown, so the ratio of (Chl + Cd)/(Po + Py) is usually used to measure the color degree of tea (Yu et al., 2019). According to this algorithm, Fuyun 6 dry tea was shown to have the highest score (1.29), followed by Mingshan 131(1.0043), Zhenong 117(0.83), Echa 1(0.69), and Echa 10 (0.57), which was consistent with the sensory evaluation results. β-carotene and lutein are also the structural and functional components of chlorophyll, thus affecting the color of green tea (He, Zhao, Zhang, Sun, & Li, 2019). Moreover, the five varieties showed no significant difference in the content of β-carotene, while Echa 10 and Zhenong 117 were higher than the other varieties in the content of lutein. Collectively, Fuyun 6 had the best dry tea color, probably due to the relatively high content of chlorophyll and chlorophyllide. Echa 10 had a darker dry tea color, probably due to the production of more pheophytin and lutein during processing, which can be improved through process optimization. Studies have shown that greenness can be maintained by reducing the conversion rate of chlorophyll through adjusting the fixation method, rolling strength, and drying temperature (Dong et al., 2018).

**Table 1:** Contents of chlorophyll for the five varieties of green tea.

	Echa 1	Echa 10	Zhenong 117	Mingshan 131	Fuyun 6
Chlorophyllide b	0.0092 ± 0.0016a	0.0048 ± 0.00076b	0.0048 ± 0.00017b	0.0063 ± 0.0021ab	0.0088 ± 0.0019a
Chlorophyllide a	0.0070 ± 0.0010b	0.0068 ± 0.00045b	0.0067 ± 0.0012b	0.0064 ± 0.0010b	0.011 ± 0.00041a
Pheophorbide b	0.018 ± 0.0044bc	0.032 ± 0.0031a	0.023 ± 0.0012b	0.013 ± 0.0023c	0.033 ± 0.0026a
Pheophorbide a	0.0035 ± 0.00030c	0.0027 ± 0.00059 cd	0.052 ± 0.00038b	0.0015 ± 0.00027d	0.013 ± 0.0010a
Lutein	0.33 ± 0.018b	0.47 ± 0.039a	0.48 ± 0.0065a	0.25 ± 0.0078c	0.34 ± 0.013b
Chlorophyll *b*	0.079 ± 0.00071a	0.075 ± 0.0065ab	0.064 ± 0.0036b	0.043 ± 0.00075c	0.061 ± 0.010b
Chlorophyll *a*	0.049 ± 0.0080b	0.034 ± 0.0038c	0.054 ± 0.0033b	0.044 ± 0.0092bc	0.081 ± 0.0046a
Pheophytin b	0.019 ± 0.00082a	0.018 ± 0.0014a	0.012 ± 0.0024b	0.0067 ± 0.000094c	0.0064 ± 0.0010c
Pheophytin a	0.13 ± 0.0059b	0.16 ± 0.0063a	0.12 ± 0.0082b	0.079 ± 0.0013c	0.074 ± 0.013c
β-carotene	0.056 ± 0.0043	0.053 ± 0.0019	0.057 ± 0.0032	0.050 ± 0.0081	0.055 ± 0.0032
Total	0.68	0.85	0.82	0.50	0.68
(Chl + Cd)/(Po + Py)	0.69	0.57	0.83	1.0043	1.29

Note: The different lowercase letters in each row indicate the least significant difference (*p* less than 0.05).

### Effects of different tea tree varieties on the aroma of Enshi green tea

#### HS-SPME-GC–MS analysis

**Fig. 3:**
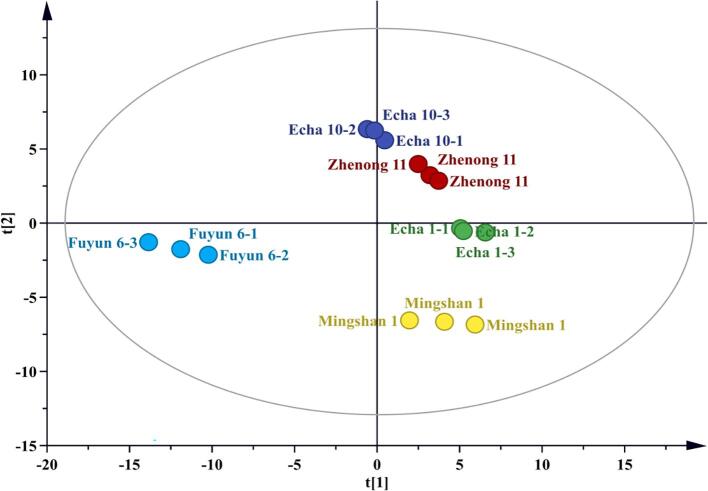
PCA statistical analysis of volatile compounds in the five different varieties.

Aroma components are essential indicators for evaluating the quality of tea (Li et al., 2022). In this study, the aroma of Enshi green tea (five tea varieties) was investigated using the HS-SPME/GC–MS technique. After peak alignment and column bleed removal, 225 compounds were initially obtained. The original data were investigated by unsupervised PCA statistical analysis and the results are shown in Fig. 3. In the score plot, the five varieties showed significant differences in volatile compounds. After further comparing the original data with the NIST2014 database and RI values, 106 volatile components were identified, including 26 alcohols, 16 alkenes, 17 aldehydes, 13 esters, 11 alkanes, 4 ketones, and 19 others (Table S1).

Among the 106 volatile components, alcohols showed the highest content, with the main substances including geraniol, benzyl alcohol, phenethyl alcohol, leaf alcohol, linalool, and their oxides, which are in line with previous studies (Zhu et al., 2018, Wang et al., 2021). For the five varieties, the content of alcohol compounds was in the order of Echa 10 (53.24%) > Zhenong 117 (52.49%) > Echa 1 (52.40%) > Fuyun 6 (44.07%) > Mingshan 131 (40.18%), with the higher alcohol content as an important potential reason for the better aroma quality of Echa 10. In Table S1, it was shown that Echa 10 was relatively high in the content of benzyl alcohol, phenethyl alcohol, geraniol and other substances; Echa 1 was relatively high in the content of leaf alcohol, linalool oxide, geraniol, nerol, and nerolidol; Zhenong 117 was higher in the content of green leaf alcohol, benzyl alcohol and linalool; Mingshan 131 was high in the content of benzyl alcohol and phenethyl alcohol. In contrast, Fuyun 6 showed a relatively low content of alcohols. Alcohol compounds are important aroma components of green tea. Many studies have shown that the main aroma compounds in green tea are linalool, benzyl alcohol, geraniol, etc. which impart different types of aroma, such as linalool with a floral, fruity, and woody aroma (Li et al., 2018, Zhu et al., 2018), geraniol (fruity, floral, sweet) (Zeng, Watanabe & Yang, 2019), benzyl alcohol and phenylethyl alcohol mainly with a sweet and citrus aroma (Li et al., 2018, Ho et al., 2015), and leaf alcohol with a fresh green leaf aroma (Guo, Ho, Schwab & Wan, 2021), which may be involved in the formation of the floral, fresh and chestnut aromas of tea.

For olefinic compounds, β-cadinene showed the highest content, followed by α-cubebene, γ-erpinene, α-farnesene, etc. In this experiment, Echa 1 was significantly higher than the other varieties in the content of β-cadinene, α-cubebene, and α-farnesene. Studies have shown that β-cadinene generally has a herbal fragrance (Wu, Lv, Lian, Wang, Gao & Meng, 2016), α-cubebene has woody, fruity and floral characteristics (Xu, Wang, & Zhu, 2021), and α- farnesene, as the main stress product, imparts a floral fragrance (Li et al., 2022). In the five varieties, Echa 1 showed a high content in these three substances, which may promote its aroma characteristics. Except for the content of β-cadinene, the five varieties showed significant differences in the other alkenes compounds. For example, Echa 10 was relatively high in the content of *trans*-2-undecenal and 1-tridecene, and Mingshan 131 was relatively high in the content of α-gurjunene and cedrene. However, Fuyun 6 is relatively lower than the other varieties in the content of most alkenes compounds. The above results may explain the difference between the five varieties in aroma quality, and Fuyun 6 has a lower content of alkenes compounds, resulting its poor performance in aroma evaluation.

Among the 16 aldehyde compounds, the substances with a higher content include nonanal, heptanal, benzaldehyde, phenylacetaldehyde, and citral (Table S1), which are commonly found in green tea (Wang, Cao, Yuan & Guo, 2021), and work together to form the aroma of tea. Comparison of the content of aldehydes found that Zhenong 117 was higher than the other four varieties in most of aldehydes, particularly phenylacetaldehyde, benzaldehyde, decanal, citral, and nonanal. Studies have shown that a low concentration of nonanal has a rose fragrance (Xu et al., 2021), citral (a fruity and herbal fragrance) (Zhu et al., 2018), benzaldehyde (a special almond odour) (Xu, Wang, Zhu, 2021) and phenylacetaldehyde (a honey smell) (Zhu et al., 2018), which contribute jointly to the formation of tea aroma. Except for Zhenong 117, the other varieties were relatively high in the content of benzaldehyde and decanal. Esters, such as acetic acid lactone, *cis*-3-hexenyl butyrate, methyl salicylate, and coumarin, are also common aroma components in green tea (Wang, Fang, Zheng, Cho & Yi, 2021), which was supported by the results of this experiment. Among the 13 detected ester compounds, dihydrokiwifruit lactone showed the highest content, and its content was significantly higher in Mingshan 131 than in the other varieties. Researchers found that acetic acid lactone has a green leaf and citrus fragrance, which can promote the formation of aroma (Zhu et al., 2018). Methyl salicylate (mint aroma) and coumarin (sweet and bitter) are more common compounds in green tea (Yu et al., 2020), and their content is relatively high in Echa 1. Additionally, the content of *cis*-3-hexenyl butyrate was significantly higher in Echa 10 than in the other 4 varieties, which mainly has a green apple-like aroma and is generally considered as the main contributor to the floral, fruity and delicate aroma of tea (Li, Luo, Ma & Zeng, 2018).

In this experiment, a total of 12 alkanes were identified, which belong to saturated hydrocarbons, and are generally considered to have little contribution to the tea flavour (Lv et al., 2014, Chen et al., 2019). The difference of ketone compounds was mainly manifested in jasmone (the highest content), which was significantly higher in Mingshan 131 than in the other varieties. A previous study has shown that jasmone has a floral and creamy fragrance, possibly related to the formation of floral and sweet aromas in green tea (Guo, Ho, Schwab & Wan, 2021).

In the 19 other compounds detected, indole and tea pyrrole showed a higher content. Indole is an important compound in the formation of tea aroma quality (Chen et al., 2019), and its content is higher in Mingshan 131. During processing, tea pyrrole is generated by Maillard reaction, imparting caramel-like aroma and sweet aroma to tea leaves (Qu, Zhu, Ai, Ai, Qiu, & Ni, 2019). In Table S1, Mingshan131 showed a significantly higher content of tea pyrrole than the other varieties, with the lowest content for Fuyun 6. Apart from the forementioned substances, we also detected 12 volatile components which failed to be identified by RI values and secondary fragments, and they were named as unknown 1–12 in the order of retention time. Their content is relatively low and the chemical structure is relatively complex. They are probably produced during the processing of green tea, and may promote or affect the formation of the aroma quality of tea. Note that among the 12 unknowns, 6 compounds are unique to Echa 10, which may play a role in the formation of its aroma quality.

In summary, the main aroma compounds in the dry tea of five varieties were alcohols, esters and aldehydes, and the five varieties showed certain differences in the aroma style, which is largely caused by the content of aroma compounds and the proportion of key substances in different tea varieties (Wang, Cao, Yuan & Guo, 2021). Combined with the sensory evaluation results, each variety is shown to be basically dominated by clean fragrance, which is closely related to phenyl alcohol, benzyl alcohol, green leaf alcohol and other important volatile components (forming the clean fragrance quality of tea (Li et al., 2018, Zeng et al., 2019) detected in these five varieties. Therefore, the content and proportion of these compounds can be assumed to determine the grade of clean fragrance characteristics in green tea.

Interestingly, the Echa 10 Enshi green tea not only has the basic clean fragrance quality, but also has a honeysuckle fragrance, enabling it to have the best performance in aroma evaluation, which agreed with a previous report (Dong et al., 2018). In previous studies, honeysuckle fragrance was defined as a clean and floral fragrance, coupled with a herbal fragrance, with the main volatile components including alcohol, aldehyde, ketone, ester, and so on. Among the volatile components, linalool, geraniol and phenylethyl alcohol are the key ingredients for the formation of the clean, floral and herbal fragrances, and substances such as hydroxycitronellal may contribute to the strength of the herbal aroma (Min and Lin, 2015, Su et al., 2020). Based on the aroma data in this experiment, Echa 10 also contained the key components (linalool, geraniol, etc.) to form the clean, floral and herbal fragrances, but showed no hydroxycitronellal (herbal fragrance boosting factor). Despite a low content, the 6 unknown substances unique to Echa No. 10 may also play a certain role in the formation of its special flavour. The special aroma type of Echa 10 is determined by the complex interaction of volatile components, but not all volatile components contribute to the formation of the final aroma quality. Therefore, in this experiment, the essential oil dilution olfactory analysis was performed to further explore the key substances and factors for the special flavour formation of Echa 10.

#### GC–MS-O analysis

Table 2 shows the GC–MS-O analysis results of odour compounds, including 8 alcohols (FD total value = 244), 5 hydrocarbons (1 4 9), 3 ketones (1 2 8), 2 esters (48), 1 aldehyde (32), and 2 others (5). Based on the results, the main contributors to the special aroma of Echa 10 are shown to be alcohols, hydrocarbons, and ketones, especially alcohols, which are similar to the results of a previous study (Guo, Ho, Schwab & Wan, 2021). The aroma substances with the highest contribution to aroma include dodecane, octadecane, phenethyl alcohol and jasmone.

**Table 2:** GC–MS-O analysis of odour compounds in green tea.

RT	RI	Substances	Aroma description	FD
		**Alcohols**		
20.55	1372.69	Leaf alcohol	grass odour	32
23.29	1477.22	2-Ethyl-1-hexanol	grass odour, green leafy smell	32
24.76	1535.34	Linalool	flowery flavour, lavender smell	16
29.41	1730.36	Linalool oxide Ⅳ	flowery flavour	32
29.87	1750.89	Linalool oxide III	flowery flavour	32
31.63	1830.84	Geraniol	flowery flavour, rose scent	32
33.13	1900.98	Phenethyl alcohol	sweet fragrance, flowery flavour	64
32.33	1863.55	Benzyl alcohol	sweet fragrance, flowery flavour	4
		Total		244
		**Hydrocarbons**		
15.76	1198.18	Dodecane	clean fragrance	64
17.35	1255.6	Styrene	chestnut flavour	1
18.52	1297.83	Tridecane	chestnut flavour, flowery flavour	16
21.21	1397.05	Tetradecane	chestnut flavour	4
30.89	1796.43	Octadecane	chestnut flavour, flowery flavour, peanut flavour	64
		Total		149
		**Ketones**		
27.65	1654.24	Acetophenone	sweet fragrance, flowery flavour	32
34.14	1950.24	Jasmone	jasmine scent	64
37.66	2129.41	Perhydrofarnesyl acetone	clean fragrance, jasmine scent, flowery flavour	32
		Total		128
		**Esters**		
12.25	1067.73	Butyl acetate	fruity flavour	32
39.23	2213.89	Methyl palmitate	ester incense	16
		Total		48
		**Aldehydes**		
24.54	1526.51	Benzaldehyde	almond fragrance, caramel scent	32
		**Others**		
34.34	1960	2-Acetyl-1H-pyrrole	roasted incense, rice smell	4
38.01	2148.13	Nonanoic acid	fat aroma	1
		Total		5

Note:FD: flavour dilution factor.

In Table 2**,** the aroma types with the highest frequency of occurrence in the Echa 10 extract are floral flavour and clean fragrance, followed by chestnut flavour and sweet fragrance, with floral flavour and clean fragrance as the dominant aroma type. There are 10 kinds of compounds related with the characteristics of floral fragrance, with the largest FD values for phenethyl alcohol and jasmone, indicating their greatest contribution to the floral characteristics of Echa 10 green tea (Feng et al., 2019, Xu et al., 2021). Secondly, the floral flavour substances with FD = 32 include geraniol, linalool oxide III, linalool oxide IV, acetophenone and perhydrofarnesyl acetone, which also play a certain role in the formation of floral flavour. There are 5 kinds of substances related with clean fragrance, with FD = 64 for octadecane and dodecane, which contribute more to the clean fragrance of tea, and the other three substances (leaf alcohol, 2-ethyl-1-hexanol, and perhydrofarnesyl acetone) also contribute to the fragrance of tea (Guo, Ho, Schwab & Wan, 2021). It is worth noting that the detected alkanes mainly exhibit a chestnut flavour, but the FD value is small for most of them, confirming their less contribution to tea flavour (Lv, Wu, Li, Xu, Liu & Meng, 2014).

In addition, octadecane (FD = 64) presents a special peanut flavour, and benzaldehyde (FD = 32) imparts almond and caramel-like fragrance. Studies have shown that benzaldehyde can also impart old white tea an obvious medicinal aroma (Chen et al., 2019). Octadecane is usually formed during the food thermal, which can produce a caramel-like fragrance and display a medicinal aroma (Li, Tian, Jiang, Lin, Liu, & Yang, 2021). Therefore, octadecane and benzaldehyde may play a crucial role in the formation of honeysuckle fragrance in Echa 10. Relevant studies have shown that the overall aroma of honeysuckle is clean fragrance and fruity flavour, coupled with a medicinal scent and fruity flavour (Min and Lin, 2015, Su et al., 2020). In this experiment, the aroma type of Echa 10 basically matched the reported aroma characteristics of honeysuckle.

### Effects of different tea tree varieties on the taste of Enshi green tea

The substances that determine the quality of a tea mainly include tea polyphenol, amino acid, soluble sugar, and aqueous extract (Li et al., 2021). Table 3 shows the contents of main biochemical components in the five different varieties of green tea. The five tea varieties showed no significant difference in the total amount of tea polyphenols. Amino acid is an important component affecting the fresh and brisk taste of tea soup (Chen et al., 2018, Yu et al., 2020). Mingshan 131 was significantly higher than the other varieties in the content of amino acid, with no significant difference among the other four varieties. Meanwhile, Echa No. 10 was significantly higher than the other varieties in the soluble sugar content. Aqueous extract was reported to affect the thickness and thinness of tea taste, whose content is significantly correlated with aroma and brew color (Hu et al., 2021). In this experiment, Echa 10 was relatively high in the aqueous extract value of dry tea. Additionally, relevant studies have shown that the phenolic ammonia ratio value can better reflect the fresh and mellow taste of a tea, and the varieties with a phenolic ammonia ratio less than 8 are usually defined as suitable for processing green teas (Chen et al., 2021). In Table 3, the phenol-ammonia ratio values are all less than 8 for the five varieties, with a relatively high value for Echa 1 (6.06) and Zhenong 117 (6.09). Studies have shown that, when the content of polyphenols and amino acids is high, the lower the the ratio of polyphenols to amino, the stronger and fresher the taste of tea soup, which endowed Mingshan No. 131 with a better taste; on the contrary, the lower the ratio of polyphenols to amino, the more astringent the taste of tea soup, which are supported by the taste evaluation results of Echa 1 and Zhenong 117 (Ai et al., 2011, Zhang et al., 2020).

**Table 3:** The contents of main biochemical components in the five different varieties of green tea (%).

Variety	Tea polyphenol	Amino acid	Soluble sugar	Aqueous extract	Ratio of polyphenols to amino	Caffeine
Echa 1	25.58 ± 1.5	4.22 ± 0.18b	3.32 ± 0.06c	57.63 ± 0.72ab	6.06	4.86 ± 0.04a
Echa 10	24.93 ± 0.52	4.24 ± 0.05b	4.01 ± 0.03a	58.26 ± 0.27a	5.88	4.22 ± 0.03c
Zhenong 117	25.31 ± 0.78	4.16 ± 0.02b	3.87 ± 0.08b	58.18 ± 0.66a	6.09	4.84 ± 0.04a
Mingshan 131	25.53 ± 0.33	4.62 ± 0.11a	3.76 ± 0.05b	56.59 ± 0.94b	5.52	4.13 ± 0.03d
Fuyun 6	24.68 ± 0.69	4.37 ± 0.22ab	3.43 ± 0.11c	57.03 ± 0.89ab	5.66	4.37 ± 0.06b

Caffeine due to its low threshold (500 μmol/L) is easily perceived by the human body, and is usually regarded as the main bitter substance of tea (Scharbert & Hofmann, 2005), so its higher content is not conducive to taste quality. In Table 3, caffeine content of green tea prepared by Echa 10 and Mingshan 131 is relatively low. The bitterness of the tea soup can reduce due to the appropriate content, so the taste scores of the two are relatively high (Fig. 1**A**). In this study, Echa 10 green tea exhibited the best performance in taste evaluation, which can be related to its reasonable ratio of polyphenols to amino and significantly higher content of soluble sugar than the other varieties. Researchers found that soluble sugar has a varying degree of effect on the sweet and mellow taste of tea soup (Wang et al., 2017). Therefore, tea polyphenol, amino acid, caffeine, and soluble sugar may make synergetic contributions to the taste quality of Echa 10.

## Conclusions

Different varieties of raw tea materials have a significant impact on the quality of green tea, mainly in terms of color, aroma, and taste. Our results show that the five tea tree varieties vary significantly in the content of chlorophyll and chlorophyllide, which are the key factors for the different colors of green needles. The dark color of some green teas can be improved through technological experiments in the future. Additionally, the five varieties of green teas showed certain differences in aroma style, and Echa 10 exhibited the best performance in aroma quality, which can be attributed to its special honeysuckle fragrance derived from the key substances, such as dodecane, octadecane, phenethyl alcohol, and jasmone. However, due to technical limitations, we are unable to study the interaction of volatile components in tea leaves currently. Future studies can focus on the aroma reorganization of Echa 10′ s honeysuckle fragrance. In taste evaluation, Echa 10 also showed the highest score, and the taste quality is mainly related to the content and ratio of tea polyphenol, amino acid, caffeine, and soluble sugar. Our integrated data indicated Echa 10 is suitable for the processing and production of high-level Enshi green tea due to its excellent taste and aroma quality.

## Declaration of Competing Interest

The authors declare that they have no known competing financial interests or personal relationships that could have appeared to influence the work reported in this paper.
