# Supplementary material for: Effects of different tea tree varieties on the color, aroma, and taste of Chinese Enshi green tea
Source: Food Chem X. 2022 Mar 22;14:100289. doi: 10.1016/j.fochx.2022.100289 (PMC8958318; doi:10.1016/j.fochx.2022.100289)
Supplement: Supplementary data 1 [file mmc1.doc]

**Table S1 The relative content of aroma components in five different green teas (ng·g-1)**

|  | Echa 1 | Echa 10 | Zhenong 117 | Mingshan 131 | Fuyun 6 |
| --- | --- | --- | --- | --- | --- |
| **Alcohols** | | | | | |
| Leaf alcohol | 162.03±31.63a | 122.4±18.89b | 170.09±29.02a | 49.52±1.08c | 46.48±3.35c |
| Benzyl alcohol | 539.6±5.63b | 662.27±63.98a | 731.27±61.59a | 643.18±106.38ab | 282.65±3.41c |
| Phenylethyl alcohol | 617.88±29.9c | 903.19±113.37ab | 872.44±150.09b | 1089.68±124.32a | 419.99±30.28d |
| Linalool | 842.73±23.41b | 532.59±19.6c | 1052.87±45.1a | 323.19±29.46d | 172.2±28.91e |
| Linalool oxide I | 385.21±41.82a | 204.6±17.55b | 211.12±10.62b | 139.38±9.16c | 54.58±10.02c |
| Linalool oxide II | 457.55±64.25a | 363.44±40.04b | 310.74±20.91b | 213.02±36c | 93.44±10.9d |
| Linalool oxide III | 814.67±58.28a | 323.38±60.49b | 354.08±63.62b | 728.69±72.16a | 173±25.89c |
| Linalool oxide IV | 101.33±4.49a | 98.74±8.09a | 77.85±5.87b | 62.54±11.65b | 26.11±2.53c |
| Geraniol | 1541.27±61.08a | 1002.86±58.01b | 996.23±17.07b | 492.84±55.69d | 608.14±24.07c |
| Nerol | 36.17±0.89a | 20.16±2.68b | 22.66±0.94b | 8.98±0.19d | 12.41±1.27c |
| Nerolidol | 30.78±5.63a | 21.6±0.31b | 19.86±0.85b | 41.4±5.47a | 7.23±0.06c |
| cis-3-Nonen-1-ol | 8.73±0.67bc | 5.58±1.03d | 19.23±1.78a | 7.89±1.23c | 10.43±1.3b |
| trans,cis-3,6-Nonadien-1-ol | 13.35±2.31a | 3.09±0.1c | 7.27±0.16b | 7.12±0.73b | 8.18±0.88b |
| 2-Butyl-1-octanol | 22.73±2.93b | 14.85±1.08c | 13.18±1.64c | 33.49±5.7a | 9.3±1.68d |
| 1,8-Octanediol | 49.17±9.41a | 34.96±0.57b | 30.46±3.9b | 48.58±0.21a | 21.44±3.26c |
| α-Terpineol | 39.43±3.16b | 31.68±0.57c | 55.32±4.18a | 14.72±0.67d | 8.21±1.16e |
| (±)-Myrtenol | 16.7±3.2bc | 12.63±1.85c | 17.72±1.78b | 22.72±2.05a | 16.9±2.28b |
| 1-Naphthalenol | 21.53±2.89a | 9.97±0.33b | - | 11.04±1.64b | 4.77±0.44c |
| 1-Tetradecanol | 19.33±1b | 21.83±2.85b | 32.85±5.58a | - | 6.15±0.69c |
| 2-Hexadecanol | 6.85±1.33b | 5.34±0.46b | 6.68±1.56b | 9.25±0.24a | 4.57±0.46c |
| (+)-Cedrol | 16.78±2.07b | 10.62±0.89d | 39.61±1.18a | 15.24±2.79bc | 11.02±1.9cd |
| 2-Ethylhexanol | 8.62±0.4a | 8.29±0.78a | 8.92±1.57a | 8.2±0.91a | 3.89±0.7b |
| 3,7,11-Trimethyldodecan-1-ol | 7.44±1.09b | 3.29±0.07c | 6.5±0.9b | 12.52±3.3a | 2.87±0.15c |
| (9Z)-hexadec-9-en-1-ol | 18.5±0.13a | 14.7±1.79b | 16.53±1.74ab | 14.79±0.65b | 2.98±0.36c |
| trans-1-Methyl-4-cyclohex-2-en-1-ol | 8.23±1.17b | 8.52±0.11b | - | 12.38±0.73a | - |
| cis-6-(Isopropyl)-3-methylcyclohex-2-en-1-ol | 6.48±1.42b | 7.3±0.86ab | 6.61±0.96b | 8.74±0.78a | 4.72±0.35c |
| **Alkenes** | | | | | |
| β-Myrcene | 41.15±3.64a | 21.22±2.44c | 33±3.92b | 25.32±3.44c | 26.61±3.81c |
| α-Copaene | 77.35±5.02a | 36.83±3.88c | 24.1±2.72d | 65.98±5.93b | 14.38±0.86e |
| α-Gurjunene | 14.82±0.16b | 6.1±0.45d | 14.89±1.12b | 40.8±2.83a | 10.39±0.66c |
| β-Caryophyllene | 13.53±3.83a | 6.02±0.96c | 2±0.13d | 8.66±1.16bc | 12.32±0.85ab |
| Cedrene | 47.54±0.77b | 16.6±1.85d | 37.88±3.11c | 99.11±6.29a | 32.12±3.92c |
| β-Cubebene | 44.28±2.95a | 2.95±0.36c | - | 29.03±1.93b | - |
| α-Caryophyllene | 5.68±0.6a | 0.87±0.11b | - | - | - |
| α-Selinene | 61.93±12.05a | 14.95±2.36c | 15.39±0.99c | 36.96±1.71b | 5.53±0.61d |
| (+)-Valencene | 13±0.09 | - | - | 12.29±2.12 | - |
| α-Farnesene | 40.2±5.28a | 15.86±0.92c | 30.77±0.35b | 35.96±1.48a | 10.42±1.15d |
| β-Cadinene | 697.44±128.98a | 133.85±3.25c | 141.32±17.99c | 400.38±66.6b | 23.72±1.21d |
| (-)-Calamenene | 64.74±3.16a | 16.76±2.39c | 22.15±2.25b | 69.93±6.54a | 8.13±0.23d |
| 1-Tridecene | - | 10±1.75a | 9.51±0.57a | 9.68±1.45a | 6.31±0.46b |
| γ-Terpinene | 60.96±0.81b | 52.73±7.82b | 70.84±11.89b | 97.85±5.73a | 46.77±3.01c |
| 3-Isobutyl-cyclohexene | 10.41±0.51a | 10.15±0.64a | 8.21±0.3b | 11.22±1.21a | 5.45±0.38c |
| (-)-α-Cubebene | 243.06±42.1a | 49.75±0.99c | 34.77±1.36d | 169.03±26.62b | 17.81±1.29e |
| **Aldehydes** | | | | | |
| Tetradecanal | 21.68±1.69a | 10.48±1.48b | 10.18±1.25b | 12.03±0.51b | 4.26±0.22c |
| Pentadecanal | 9.48±1.33a | 8.28±0.5a | 8.25±1.17a | - | 3.14±0.4b |
| Heptanal | 5.28±0.11c | 5.51±0.94bc | 17.85±4.87a | 7.12±1.34bc | 9.8±1.34b |
| (E)-2-Heptenal | 6.13±1.09c | 12.3±1.37a | 6.02±0.56c | 9.08±1.64b | 3.61±0.55d |
| Benzaldehyde | 69.98±4.58c | 114.38±1.96a | 114.85±17.61a | 89.81±12.5b | 57.31±0.95c |
| Octanal | 52.9±0.45b | 66.75±4.05a | 55.57±1.95b | 55.61±8.11b | 29.26±2.52c |
| (E,E)-2,4-Heptadienal | 11.47±1.62b | 17.61±2.19a | 20.2±1.77a | 9.6±1.37b | 10.43±1.79b |
| Phenylacetaldehyde | 15.39±0.72d | 51.12±2.14c | 118.18±20.38a | 66±10.6bc | 70.64±0.03b |
| Nonanal | 178.07±12.9c | 230.5±3.43b | 306.61±13.85a | 254.25±40.29b | 217.16±18.91b |
| (2E)-2-Nonenal | 19.55±1.4b | 25.69±4.55a | 31.42±2.02a | 30.27±3.68a | 26.72±3.66a |
| Decanal | 118.47±20.43b | 188.77±22.48a | 234.79±46.38a | 130.73±24.59b | 99.07±9.2b |
| (E)-2-Decenal | 73.79±14.18a | 34.37±2.35c | 46.5±3.31b | 36.63±2.37c | 25.56±2.35d |
| Citral | 24.94±3.02a | 15.15±0.06c | 21.92±0.93a | 22±2.47a | 19.13±2.25a |
| Lauryl aldehyde | 19.26±0.88b | 27.5±4.62a | 29.18±3.94a | 16.42±1.12c | 8.59±0.93d |
| 2-Butyl-2-octenal | - | - | 8.41±1.19c | 14.88±1.84a | 9.38±1.57b |
| Dolichodial | 31.8±4.82b | 19.38±3.41cd | 24.18±2.17c | 45.76±4.55a | 16.17±2.27d |
| trans-2-Undecenal | 31.42±0.98a | 30.94±0.18a | 33.57±4.21a | 24.75±1.38b | 14.47±1.2c |
| **Esters** | | | | | |
| (7Z)-6,10-dimethylundeca-5,9-dien-2-one | 22.65±3.3b | 8.61±0.35c | 21.52±0.88b | 35.77±5.25a | 31.42±5.33a |
| Acetic acid lactone | 374.19±18.93c | 254.94±18.79d | 418.12±53.61bc | 696.39±46.45a | 430.87±26.1b |
| (3Z)-3-Hexen-1-yl benzoate | 18.01±0.26a | 5.63±0.26c | 11.91±1.06b | 13.71±3.13b | 6.5±0.81c |
| Ethyl laurate | 10.13±1.27a | 9.22±0.02a | 9.83±1.11a | 9.25±0.91a | 3.88±0.29b |
| Methyl octadeca-2,5-diynoate | 12.22±0.09a | 3.13±0.39b | - | - | 0.96±0.03c |
| Ethyl tetradecanoate | - | 5.52±0.93a | - | - | 3.95±0.54b |
| Didodecyl benzene-1,2-dicarboxylate | 16.27±0.12c | 21.75±1.7a | 23.79±0.81a | 18.13±0.1b | 12.1±0.85d |
| Allyl 2-ethylbutyrate | - | 13.01±0.36a | 6.47±1.28b | - | 4.49±0.64c |
| Cis-3-Hexenyl butyrate | 55.26±1.87b | 59.53±0.18a | 47.12±1.95c | 31.05±5.41d | 28.68±1.61d |
| Methyl salicylate | 249.91±3.18a | 94.88±19.04bc | 246.84±11.33a | 75.57±13.73c | 118.3±5.07b |
| Cis-3-Hexenyl 2-Methylbutanoate | 9.76±1.05a | 5.88±0.27d | 8.98±0.81a | 7.65±0.97c | 6.63±1.07b |
| (Z)-3-Hexen-1-yl hexanoate | 169.86±12.82a | 82.75±2.04b | 150.43±11.71a | 62.8±4.03c | 53.5±9.06c |
| Coumarin | 54.79±12.96a | 14.15±2.43c | 22.4±3.11b | 9.37±1.27d | 7.13±0.15e |
| **Alkanes** | | | | | |
| Tetradecane | 76.54±23.11a | 42.59±3.54b | 49.4±6.22b | 75.24±9.5a | 15.76±2.04c |
| 4-Methyltetradecane | 8.06±1.57b | 5.55±0.14c | 8.09±1.39b | 15.61±1.39a | 4.58±0.43d |
| Tridecne, 6-propyl | 12.76±0.28b | 8.79±0.06c | - | 26.31±2.54a | 5.9±1.52d |
| Hexadecane | 47.57±1.78b | 27.62±1.6c | 61.68±5.43a | 59.56±7.7a | 12.57±1.19d |
| Heptadecane | 31.1±5.71ab | 21.48±0.05c | 39.07±2.66a | 31.41±0.36b | 15.98±0.74d |
| 2,6,10-trimethyltetradecane | - | 6.47±0.14 | 12.9±0.94 | 21.19±1.93 | 4.53±0.38 |
| Pentadecane | 39.52±2.36b | 21.26±1.21c | 46.01±3.53ab | 48.7±8.59a | 10.86±0.83d |
| Dodecane | 158.08±25.39b | 217.75±15.1a | 239.25±7.43a | 177.27±29.16b | 60.63±4.07c |
| Tridecane | 166.07±31.51b | 196.22±31.98b | 246.28±4.48a | 177.13±22.52b | 64.72±4.23c |
| Cyclododecane | - | 10.84±0.79b | 12.2±0.9b | 21.19±2.29a | 19.09±3.02a |
| 1-Undecyne | 10.6±0.24d | - | 16.16±2.02c | 24.59±0.71b | 35.13±2.4a |
| **Ketones** | | | | | |
| Jasmone | 888.08±28.47b | 642.98±127.72c | 698.34±11.33c | 1526.68±11.46a | 404.87±11.63d |
| 6,10,14-Trimethyl-2-pentadecanone | 30.77±5.3b | 57.41±1.97a | 41.76±5.38b | 18.32±0.63c | 13.56±0.47d |
| 5-Ethyl-6-methyl-3-hepten-2-one | - | - | - | - | 12.38±0.96 |
| 3,5-Octadiene-2-one | 159.44±6.77a | - | 176.67±14.01a | - | 96.43±16.9b |
| **Others** | | | | | |
| Indole | 252.94±1.48c | 221.72±31.89c | 398.07±27.27b | 459.28±36.34a | 130.6±6.61d |
| Phenol | 19.35±3.12a | 10.48±0.74b | 5.37±0.23c | 9.63±0.26b | 9.5±1.19b |
| Caryophyllene oxide | - | 2.89±0.06a | 8.36±0.71b | - | 7.2±1.04b |
| 1-Ethyl-1h-pyrrole-2-carbaldehyde | 111.53±10.05b | 113.56±14.95b | 118.39±6.45b | 160.52±22.41a | 40.91±2.97c |
| 3,5-Di-tert-butylcatechol | 18.65±0.14b | 32.35±1.39a | - | 17.72±1.18b | 10.66±0.43c |
| 2-Butylthiolane | 115.95±7.87b | 74.33±10.78d | 93.77±16.1cd | 163.29±12.41a | 99.22±11.99bc |
| 2,3-Dihydrobenzofuran | 81.84±3.2b | 62.29±2.25c | 57.83±9.16c | 118.56±13.23a | 38.36±4.96d |
| Unknown 1 | - | 15.53±0.55b | 14.83±1.49b | 25.78±1.21a | - |
| Unknown 2 | - | 4.7±0.69 | - | - | - |
| Unknown 3 | - | - | 11.74±0.73a | 9.06±1.27b | 7.31±0.62c |
| Unknown 4 | - | 9.62±1.55b | 13.68±0.81a | 7.84±0.58c | 9.34±1.74b |
| Unknown 5 | - | 4.37±0.09 | - | - | - |
| Unknown 6 | - | 54.94±0.33 | - | - | - |
| Unknown 7 | 26.28±2.48a | 5.11±0.4b | - | - | 2.2±0.11c |
| Unknown 8 | 122.06±24.2a | 24.32±3.72c | - | 56.31±5.77b | 12.96±0.67d |
| Unknown 9 | - | 2.01±0.23 | - | - | - |
| Unknown 10 | - | 38.75±0.71 | - | - | - |
| Unknown 11 | - | 7.65±0.36 | - | - | - |
| Unknown 12 | - | 3.05±0.13a | - | - | 0.79±0.03b |

Note: Different lowercase letters in each row indicate significant difference at p < 0.05; -: not detected.
